# Surface-Enhanced Raman Scattering Spectroscopy Combined With Chemical Imaging Analysis for Detecting Apple Valsa Canker at an Early Stage

**DOI:** 10.3389/fpls.2022.802761

**Published:** 2022-03-04

**Authors:** Shiyan Fang, Yanru Zhao, Yan Wang, Junmeng Li, Fengle Zhu, Keqiang Yu

**Affiliations:** ^1^College of Mechanical and Electronic Engineering, Northwest A&F University, Yangling, China; ^2^Key Laboratory of Agricultural Internet of Things, Ministry of Agriculture and Rural Affairs, Yangling, China; ^3^Shaanxi Key Laboratory of Agricultural Information Perception and Intelligent Service, Yangling, China; ^4^College of Plant Protection, Northwest A&F University, Yangling, China; ^5^School of Computer and Computing Science, Zhejiang University City College, Hangzhou, China

**Keywords:** apple Valsa canker, early detection, Surface-Enhanced Raman Scattering, chemical imaging, machine learning

## Abstract

Apple Valsa canker (AVC) with early incubation characteristics is a severe apple tree disease, resulting in significant orchards yield loss. Early detection of the infected trees is critical to prevent the disease from rapidly developing. Surface-enhanced Raman Scattering (SERS) spectroscopy with simplifies detection procedures and improves detection efficiency is a potential method for AVC detection. In this study, AVC early infected detection was proposed by combining SERS spectroscopy with the chemometrics methods and machine learning algorithms, and chemical distribution imaging was successfully applied to the analysis of disease dynamics. Results showed that the samples of healthy, early disease, and late disease sample datasets demonstrated significant clustering effects. The adaptive iterative reweighted penalized least squares (air-PLS) algorithm was used as the best baseline correction method to eliminate the interference of baseline shifts. The BP-ANN, ELM, Random Forest, and LS-SVM machine learning algorithms incorporating optimal spectral variables were utilized to establish discriminative models to detect of the AVC disease stage. The accuracy of these models was above 90%. SERS chemical imaging results showed that cellulose and lignin were significantly reduced at the phloem disease-health junction under AVC stress. These results suggested that SERS spectroscopy combined with chemical imaging analysis for early detection of the AVC disease was feasible and promising. This study provided a practical method for the rapidly diagnosing of apple orchard diseases.

## Introduction

Apple Valsa canker (AVC), caused by fungus *Valsa mali*, is a severe apple tree disease resulting in serious economic losses in Southeast Asia and China (Wang et al., 2011). Commonly, AVC is mainly found by the characteristics of canker, infected tissue softening, outflowed light brown water stain, sunken or cracked on trunks at the early infected stage (Zang et al., 2012). The fungal pathogen mainly infected the subcutaneous phloem through the wounded bark tissue at the initial infected stage. After infection, fungus hypha colonized the phloem tissues, leading to severe tissue cell death (Suzaki, 2008). What’s more, plant protection experts have proved that the fungus *Valsa mali* can survive in weak and dead tissues of the apple trees for more than 1 year before appearing visible symptoms (Meng et al., 2019). For example, Zang et al. (2012) found that more than 50% of apple orchards existed fungus *Valsa mali* in symptomless apple tree tissues. However, when visible symptoms appear, it is challenging to prevent AVC from spreading throughout the orchard by conventional treating methods such as spraying fungicides, manually removing the diseased areas, and pruning the dead branches. Unfortunately, there were no adequate methods for AVC treatment due to the complicated pathogenic mechanism so far. Thus, early detection of the infected trees is necessary to prevent the rapid development of the disease in orchards.

There are various molecular biology methods, including Enzyme-Linked ImmunoSorbent Assay (ELISA) and Polymerase Chain Reaction (PCR), were developed for the isolation and identification of pathogenic (Liu et al., 2015; Golhani et al., 2018). ELISA kits have been widely utilized thanks to the low cost, but are ineffective for detecting symptomless tissue (Fang and Ramasamy, 2015), while PCR is an effective detection method. Zang et al. (2012) developed a nested PCR assay to detect the presence of *Valsa mali* in apple trees and achieved an accuracy of 64.7%. However, DNA deriving from the woody plant tissues contained PCR inhibiting compounds and could affect the accuracy of PCR reaction (Martinelli et al., 2015). What’s worse, a well-equipped laboratory and experienced personnel are also required, which was not feasible for on-site detection using the PCR (Okiro et al., 2019). Therefore, it is of great significance to develop a fast, non-destructive and economical method for accurate detection of AVC.

Reported studies have demonstrated that advanced non-invasive measuring technologies, such as RGB image processing (Cruz et al., 2019; Hu et al., 2020), dielectric spectrum (Khaled et al., 2018), laser scanning (Khairunniza and Vong, 2014), and spectroscopic methods (Ranulfi et al., 2016; Dou et al., 2021) have a massive amount of potential for diagnosing tree diseases. Among them, the spectroscopy technique is powerful for quality and safety inspection due to the character of simplicity, rapidity, and affordability, which makes it indispensable in tree disease detection. Raman spectroscopy (RS) is a non-invasive, rapid, and high throughput spectroscopic technique (Farber et al., 2020; Huang et al., 2020; Zhao et al., 2021). Raman shift is only related to the vibration frequency of the molecular functional group, but not to the incident light. Therefore, each sample’s the Raman “fingerprint” of each sample is unique (Fang et al., 2021). Significantly, RS could provide essential information related to the biochemical composition of the tree tissue cell, such as protein, polysaccharide, and lipid. Neither symptomatic nor asymptomatic trees, these biochemical compositions are significantly different between diseased and healthy tissue. These compositions changes can be reflected in Raman shifts or intensity changes of specific Raman bands assigned to those molecules. Therefore, RS provides an accessible way to identify subtle changes in the molecular compounds, which offers theoretical evidence for detecting tree diseases. Vallejo et al. (2016) investigated the application of RS combined with statistical analysis for detecting citrus Huanglongbing (HLB) infection in the field, and a good result was obtained with an overall classification accuracy of about 89.2%. Sanchez et al. (2019b) readily distinguished between healthy and early-HLB citrus trees using a handheld Raman system and achieved an accuracy of 94%. In their following study, Sanchez et al. (2019a) demonstrated that utilizing a handheld Raman spectrometer in combined with chemometric analyses enabled the detection and identification of the secondary disease on HLB-infected orange trees. Those researches indicated that the RS technique combined with chemometrics methods could detect diseased trees.

However, RS is frequently interfered by fluorescence caused by chromophores in plant tissue, and compositional changes under disease stress may lead to Raman band broadening or drift (Mukherjee et al., 2017; Petrov, 2017). This drawback may lead to significant deviations in the biochemical composition analysis of RS data. Surface enhanced Raman scattering (SERS) spectroscopy, based on the improvement of traditional RS, uses certain metallic nano-substrates such as gold or silver nanoparticles (AgNPs) to enhance signals under low laser powers, which maximizes fluorescence suppression. Meanwhile, the Raman system combined with the micro-imaging technology allows for scanning micron-scale Raman collection points (e.g., one-micron pixel) (Li X. L. et al., 2019), which offers chemical information on the constituents at a high spatial resolution *in situ*. Qin et al. (2011) developed a Raman chemical imaging system to visualize the internal distribution of lycopene in postharvest tomatoes and established a Raman chemical image to visualize the spatial distribution of lycopene at different stages of maturity. Yang et al. (2018) used a Raman imaging system to detect the spatial distribution of chemical components in maize seeds. These studies manifested that Raman chemical imaging has great potential in the visualizing of plant tissue components.

Therefore, this study aimed to develop a fast, non-invasive, and *in situ* diagnosis method for detecting AVC at early infection stages using SERS combined with micro-imaging technology. The main objectives are to: (1) Optimize experimental conditions (i.e., laser intensity and exposure time) for obtaining valid SERS micro-imaging data, including Synthesis and SERS AgNPs characterization; (2) Establish optimal discriminative models for detecting AVC in early infection stages based on machine learning algorithms; (3) Generate micro-distribution maps of cellulose and lignin at the disease-health junction of the tree phloem tissues to reveal the dynamic development characteristics of the disease.

## Materials and Methods

### Fungal Culture and Sample Inoculation

The fungus *Valsa mali* stored at −80°C in an ultra-low temperature refrigerator were inoculated onto potato dextrose agar (PDA) medium. The 2-year-old apple branches (*Malus domestica* cv. Fuji) were collected from the Economic Tree Garden of Northwest A&F University. The selected branches were pruned into 15 cm segments, and the surface of the branches was disinfected with 75% alcohol for 15 min. Then, they were cleaned with sterile water three times until there was no odor. The ends of the branches were sealed with a wet skimmed cotton to keep them fresh, followed by punching holes in the branches with a hole puncher (hole diameter 5 mm). The activated *Valsa mali* fungus was inoculated on the wounds of apple branches with two points on each branch. After inoculation, the branches were transferred to a 25°C incubator for further incubation.

### Synthesis and Surface-Enhanced Raman Scattering Silver Nanoparticles Characterization

In the present research, AgNPs were synthesized by using the Lee–Meisel method. The synthesis steps were as follows: AgNO_3_ (36 mg) was dissolved in 200 ml of ultrapure water and boiled quickly. A solution of 1 wt.% trisodium citrate (6 mL) was charged to the reaction solution and was held on boiling for 25 min accompanied by stirring at 200 rpm. After cooling to room temperature, we pour the AgNPs solution into a centrifuge tube and store it away from light. The chemical reaction equation is as follows:


$$4\,A\,g^{+}+C_{6}\,H_{5}\,O_{7}\,N\,a_{3}+2\,H_{2}\,O=4\,A\,g+C_{6}\,H_{5}\,O_{7}\,H_{3}+3\,N\,a^{+}+O_{2}$$

Subsequently, the prepared AgNPs were characterized to verify their validity. The morphology of the AgNPs was measured by Tecnai G2 transmission electron microscopy (FEI Inc., Hillsboro, OR, United States). The UV-Vis absorption spectra of the AgNPs were measured using Lambda 35 Spectrophotometer (PerkinElmer Inc., Waltham, MA, United States). The Raman spectra of the AgNPs were collected by DXR3xi Raman micro-imaging spectrometer (Thermo Fisher Scientific Inc., Waltham, MA, United States).

### Surface-Enhanced Raman Scattering Spectroscopy Acquisition

First, branches were removed from the incubator, and the inoculation points on the phloem were scraped with a knife as the samples. Each sample placed on a glass slide was dripped with the AgNPs. Then, each sample was placed on the automatic stage and aligned with a Raman laser using a 10x/0.25 NA magnification objective lens for SERS imaging collection using a DXR3xi Raman micro-imaging system (Thermo Fisher Scientific Inc., Waltham, MA, United States). Specific parameters were to: the excitation wavelength was 785 nm; the collected spectral range was 300–3,000 shift/cm^–1^; the laser intensity was 2.6 mW; the exposure time was 0.00285 s (350 Hz); the number of scanning was 40.

For spectral imaging in the *x* and *y* directions, the samples were scanned point by point in 2 μm steps. It should be noted that no destructive effects of the laser on the samples were observed. Routinely, before starting the Raman measurements, the calibration procedure that came with the instrument was executed automatically. At this time, the software interface displayed “Performing automatic *X* axis calibration.” The data acquisition software OMNICxi v1.6 was used to adjust the acquisition parameters.

### Spectral Data Processing and Analysis

#### Spectra Preprocessing

Background noises and baselines were generated during the acquisition of the SERS spectra, which seriously impaired the interpretability of the spectra. Meanwhile, these noises and baselines would also reduce the simplicity and robustness of the calibration model built on these spectra. Therefore, selecting the optimal pretreatment method was necessary to improve the spectral quality. In this study, spectral curves were first extracted for each pixel point of the imaging data before spectra preprocessing. Then, the spectral data were preprocessed with three algorithms to eliminate noise and correct the baseline background. These three algorithms include the multiple spectral baseline correction (MSBC), the asymmetric least squares (AsLS), and the adaptive iterative reweighted penalized least squares (air-PLS). Subsequently, the advantages and disadvantages of the three algorithms were compared using the correlation analysis method.

The AsLS method, proposed by Eilers (2003, 2004), is a classical baseline correction algorithm that combined a smoother with the asymmetric weighting of deviations from the smoothed trend to form an effective baseline estimation method. The MSBC method, proposed by Peng et al. (2010), is an improved approach based on the AsLS algorithm. The MSBC method learns baselines that perform well on the corresponding spectra and then “co-regularize” the selection by correcting inconsistencies between the spectra. Air-PLS is an improvement approach based on the weighting of the original model by the weighted least squares method. The light environment is automatically subtracted by meaning the iterative regression, and the background is deducted (Baek et al., 2015).

#### Optimal Variables Selection and Dimension Reduction

Multivariate calibration methods in chemometrics aim to construct relationships between variables and properties of interest to make a classification model. However, with the redundant spectral variables, data usually included some noise and unnecessary information, which rendering unreliable predictive properties. Therefore, optimal variables selection and dimension reduction have been used to address these problems.

Principal component analysis (PCA) can replace the original variables with a few principal components with significant deviation to reduce the original high-dimensional variable space (Dong et al., 2014). In addition, competitive adaptive reweighted sampling (CARS) and random frog (RFrog) algorithms were combined to select the optimal variables associated with the predicted properties and exclude the interference of unrelated variables. The CARS algorithm used exponentially decreasing function (EDF) as a selection strategy to select critical variables based on adaptive reweighted sampling competitively (Li et al., 2009; Li Q. Q. et al., 2019). The RFrog algorithm calculated the selection probability of each variable by moving across trans-dimensions between models, enabling the search for the optimal variable (Li et al., 2012).

#### Classification Models

BP artificial neural network (BP-ANN) (Zhang et al., 2018) is the most classical and successful neural network commonly utilized for non-linear fitting and pattern recognition. BP-ANN is a one-way multi-layer feedforward network composed of an input, hidden, and output layer. The learning process is composed of forwarding propagation of signals and back-propagation of errors.

The random forest (RForest) is a widely used machine learning algorithm, which has been successfully applied to pattern recognition (Lussier et al., 2020), and the choice appropriate number of decision trees is crucial in RForest. When the test data entered the classifier, each decision tree classified the data. Finally, the class with the most classification results from all decision trees was taken as the result.

The least squares support vector machine (LS-SVM) is a machine learning method that emerged from the statistical learning theory. LS-SVM divides the data samples into multi classes by determining a hyperplane in the input space, maximizing the separation between the classes (Lucay et al., 2020). Its vital parameter indexes are the kernel function and the corresponding parameters of this function.

Extreme learning machine (ELM) is one of the practical training algorithms for single-layer feedforward neural networks (Qiu et al., 2015). ELM has a faster training and better generalization performance than traditional machine learning algorithms and could overcome issues such as the local minimum, inappropriate learning rate, and overfitting (Wu et al., 2021). Therefore, it is widely used in the condition of classification and regression.

**FIGURE 1:**
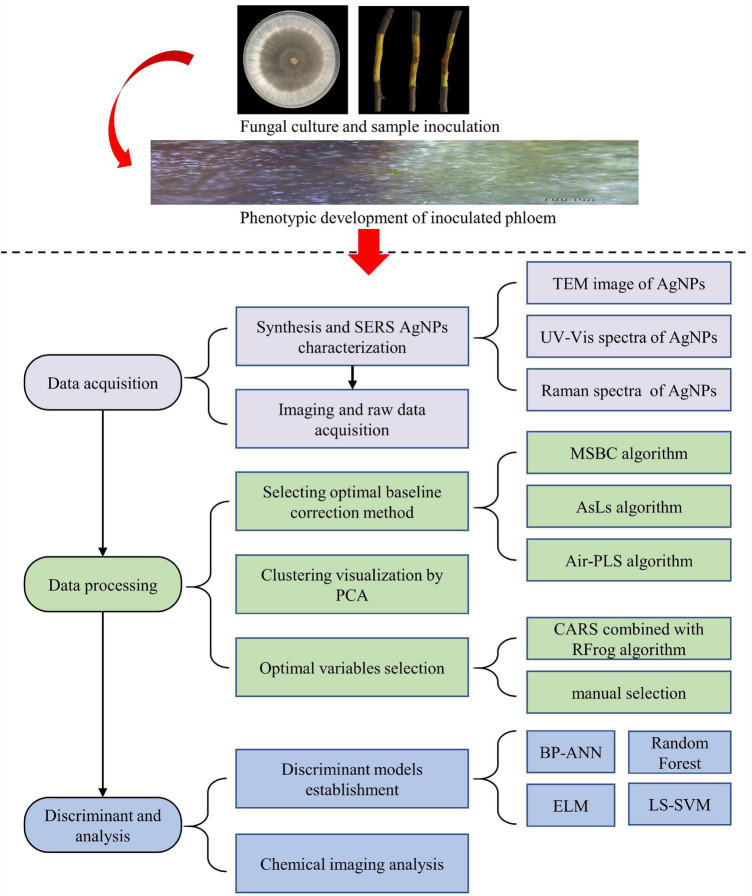
Key steps for detecting apple Valsa canker at early stage based on SERS combined with chemical imaging analysis. There were four main steps in the experiment: step 1, preparation of the samples; step 2, data acquisition; step 3, data processing; step 4, discriminant and analysis.

In summary, Figure 1 demonstrated Key steps for detecting apple Valsa canker at an early stage based on SERS spectroscopy combined with chemical imaging analysis. All procedures were written in MATLAB R2018b (The MathWorks, Natick, MA, United States) and ran on a personal computer with an Intel Core i5-9400F CPU, 16GB RAM, and a Windows 10 operating system.

## Results and Discussion

### Phenotypic Development of Healthy and Inoculated Branch

**FIGURE 2:**
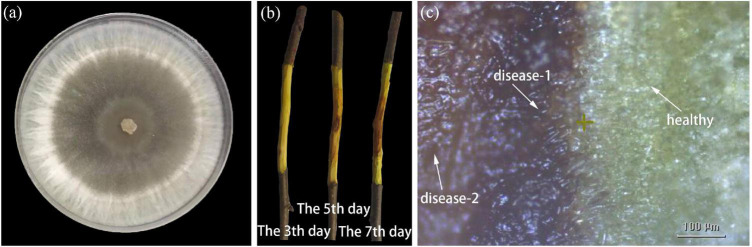
Phenotypic development of healthy and inoculated branch. **(a)** The strains of the fungus *Valsa mali* on PDA medium. **(b)** The dynamic process of the diseased phloem in the first 7 days. **(c)** Optical micrograph of the disease-health junction.

Figure 2a demonstrated the strains of the fungus *Valsa mali* on the PDA medium. The junctions of diseased and healthy tissues in the inoculated branch samples were assessed visually in the early stage of AVC disease. The bark surface of inoculated branch samples showed no visible symptoms during the first 7 days. However, the phloem inside the bark appeared with early infection symptoms. Figure 2b demonstrated the dynamic process of the diseased phloem in the first 7 days. The healthy phloem (the first 3 days) had a smooth surface and displayed tender green. The diseased phloem became rough and showed pale brown when the symptoms of mild infection were visible on the 5th day. Subsequently, the diseased phloem appeared dark brown, and the tissue was rotten on the 7th day. The infected area of the diseased phloem, centered on the inoculation site, was continuously extended outward with time. Most notably, the infection symptoms remained in the phloem and did not appear on the bark surface in the first 7 days. The phloem regions were manually labeled as healthy, disease-1 (the disease-health intersection), and disease-2 (late-disease) according to the infection progression of the pathogen. The purpose of dividing the region into three categories is to simulated the time-series dynamic process of pathogen infection (i.e., pathogen infection spread outward around the center point). In Figure 2c, the disease-health intersection of the diseased phloem was presented using optical microscopy. It can be observed that the healthy tissue appeared green with intact cellular tissue structure; The disease-1 tissue appeared dark brown, and the infected tissue outflowed light brown water stain; The disease-2 tissue was mainly characterized by canker and softened tissue.

### Surface-Enhanced Raman Scattering Silver Nanoparticles and Its Characterization

**FIGURE 3:**
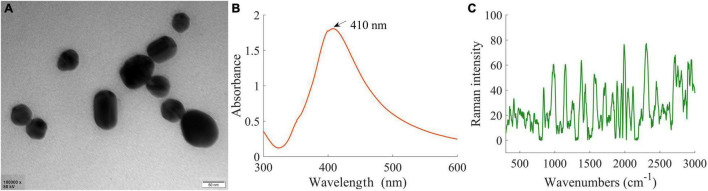
SERS AgNPs and its characterization. **(A)** Transmission electron microscopy image of AgNPs. **(B)** The UV-Vis spectra of AgNPs. **(C)** Raman spectrum of AgNPs.

The microstructure, UV-Vis spectrum, and Raman spectrum of AgNPs were analyzed to investigate the enhancement effects of the synthesized AgNPs. Figure 3A is the transmission electron microscopy (TEM) image of AgNPs, Figure 3B displays the UV-Vis spectra, and Figure 3C shows the Raman spectra.

In Figure 3A, it could be seen that the morphological character of AgNPs was very uniform in a monodisperse spherical shape. In addition, the average diameter of AgNPs was about 50 nm. As shown in Figure 3B, only one UV-Vis characteristic absorption peak (at 410 nm) corresponding to the single plasmon resonance mode was observed, and the half-peak breadth was only 90 nm. These features further indicated that the shape and size of the synthesized AgNPs were very uniform. In Figure 3C, the Raman spectrum had a faint signal, suggesting that the synthesized AgNPs themselves had no strong Raman characteristic peaks and did not have an interferential effect on experimental results. Therefore, the synthesized AgNPs were suitable as SERS substrate to detect branch samples in this research.

### Overview of Surface-Enhanced Raman Scattering Spectra

**FIGURE 4:**
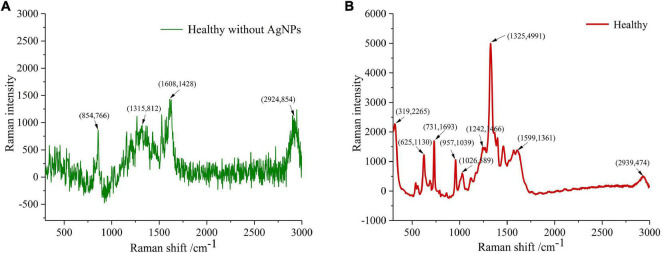
The spectrum of healthy tissue samples, with and without AgNPs, respectively. **(A)** Raman spectra peaks of healthy samples without AgNPs did not appeared. **(B)** The SERS characteristic peaks of healthy samples were obvious, which further proved that AgNPs was effective.

**FIGURE 5:**
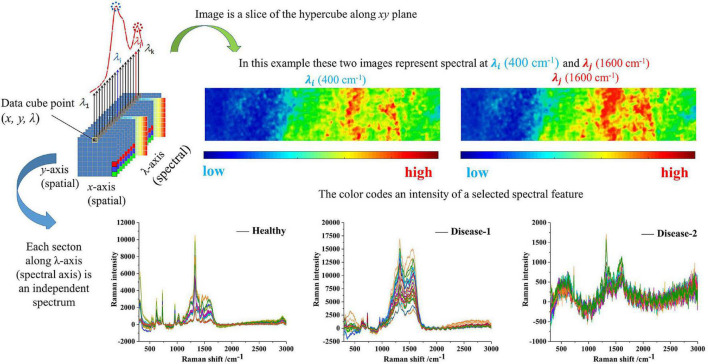
The sketch represents the basic principle of the spectral data cube and shows the raw spectra and spectral imaging of three types of samples.

Spectral imaging is capable of acquiring the spectra from a specified point at the sample surface. By adjusting the *x, y* position, acquisitions of the spectra from multiple points on the sample surface can be performed, assembling a spectral image of the sample. Figure 4 clearly showed the spectrum of healthy tissue samples, with and without AgNPs, respectively. Raman spectra peaks of healthy samples without AgNPs did not appear. The SERS characteristic peaks of healthy samples were obvious, which further proved that AgNPs were effective. Figure 5 showed the micro-spectral image of diseased phloem through pointwise scanning by Raman micro-imaging system. The spectral data were obtained by splitting each pixel point of the spectral image. All the original SERS spectra were also shown in Figure 5. The pathogenic mechanism of AVC remains poorly understood (Wang et al., 2021). On the one hand, cell wall degrading enzymes (e.g., pectinases) played an important role in the infection process (Yin et al., 2013). On the other hand, studies have shown that phloridzin in apple tissues can be degraded by AVC, and the metabolites have toxic effects on apple tissue cells (Feng et al., 2020). These researches explained why the vibration band of disease-2 is weaker than the health spectrum.

**FIGURE 6:**
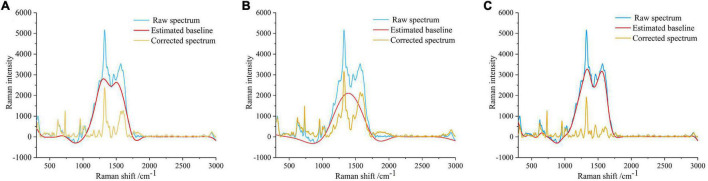
Spectral baseline correction. **(A)** Baseline correction using MSBC. **(B)** Baseline correction using AsLS. **(C)** Baseline correction using air-PLS. The blue line represents the original spectrum, the red line represents the estimated baseline, and the yellow line represents the corrected spectrum.

There was an obvious baseline offset in the disease-1 and disease-2 even after dropwise addition of the AgNPs to suppress fluorescence. Therefore, the MSBC, AsLS, and air-PLS algorithms were adopted to eliminate the disturbances of the baseline offset. The parameters for these methods were manually set to obtain the best result. For the MSBC algorithm, the parameters were set to λ = 150, μ = 8 × 10^7^, and ρ = 0. For AsLS algorithm, the parameters were set to λ = 5,000, and ρ = 0.0001. For the air-PLS algorithm, the parameters were set to λ = 150, and ρ = 0.01. The corrected spectra and the predicted fluorescence baselines were plotted in Figures 6A–C. As shown in Figure 6, the curved baselines were well-fitted and subtracted by the three algorithms. The corrected spectra showed that the baselines were pulled back to zero absorbance, the peak locations remained unchanged, and the peak shapes were more prominent, which indicated the effectiveness of the baseline correction methods.

As shown in Figure 6, many SERS peaks can be clearly observed. In detail, the peaks at 319, 957, 1,026, 1,165, 1,242, and 1,325 cm^–1^ were indicators of cellulose, corresponding to C-C-C or C-O-C skeletal bending (Szymanska et al., 2011), C-C or C-O stretching vibration (Beć et al., 2020), C-C or C-O stretching vibration (Beć et al., 2020), H-C-C or H-C-O skeletal bending (Edwards et al., 1997), C = O stretching vibration (Beć et al., 2020), and C-H bending vibration (Edwards et al., 1997), respectively. The peaks at 625, 731, 1,599, and 2,939 cm^–1^ were indicators of lignin, corresponding to skeletal bending (Agarwal et al., 2011), skeletal bending (Agarwal et al., 2011), C-C aromatic ring (Agarwal, 2006), and C-H asymmetric stretching vibration (Gierlinger and Schwanninger, 2007), respectively. The assignment of characteristic wavenumbers was presented in Table 1.

**TABLE 1:** Assignment of characteristic wavenumbers.

Wavenumber	Assignment	Biological components	References
319	C-C-C or C-O-C skeletal bending	Cellulose	Szymanska et al. (2011)
625	Skeletal bending	Lignin	Agarwal et al. (2011)
731	Skeletal bending	Lignin	Agarwal et al. (2011)
957	C-C or C-O stretching vibration	Cellulose	Beć et al. (2020)
1,026	C-C or C-O stretching vibration	Cellulose	Beć et al. (2020)
1,165	H-C-C or H-C-O skeletal bending	Cellulose	Edwards et al. (1997)
1,242	C = O stretching vibration	Cellulose	Beć et al. (2020)
1,325	C-H bending vibration	Cellulose	Edwards et al. (1997)
1,599	C-C aromatic ring	Lignin	Agarwal (2006)
2,939	C-H asymmetric stretching vibration	Lignin	Gierlinger and Schwanninger (2007)

### Selecting Optimal Preprocessing Method

**FIGURE 7:**
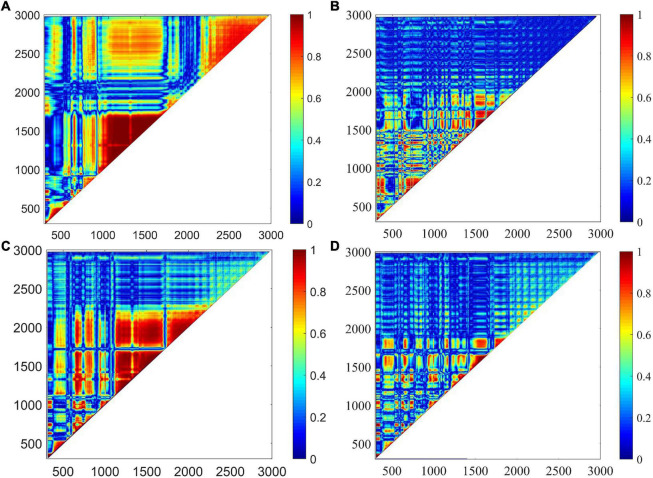
The correlation between the corrected variables was plotted. **(A)** High correlations were found among original spectral variables. **(B)** Correlations were noticeably declined using MSBC. **(C)** Correlations were noticeably declined using AsLS. **(D)** Correlations were noticeably declined using air-PLS. However, the air-PLS algorithm has the best elimination effect.

The correlation analysis method was adopted to select the best preprocessing algorithms. The correlation between the corrected variables was plotted in Figure 7. Significantly, the regions close to the line *y* = *x* had a correlation coefficient of 1, indicating that the original spectra were greatly disturbed by the baseline offset. This high degree of collinearity would cause adverse effects on classification analysis. Comparing Figures 7B–D with Figure 7A, the regions with a high degree of collinearity have a noticeable decrease, and most of the spectral variables had low correlation with others except in the spectral ranges of 300–400, 640–880, and 1,490–1,970 cm^–1^. In addition, the proportion of pixel points with values greater than 0.6 to the total pixel points was calculated, and the proportions were 0.35, 0.09, 0.24, and 0.07, respectively. The AsLS method failed to effectively fit the baseline at 1,200–1,600 cm^–1^, resulting in a relatively poor result of baseline correction. This result indicated that the MSBC and air-PLS baseline offset elimination strategies could greatly reduce the high correlation levels among spectral variables, and especially, the air-PLS algorithm had the best elimination effect. Therefore, the spectra corrected by the air-PLS algorithm were used for further analysis.

### Clustering Visualization by Principal Component Analysis

**FIGURE 8:**
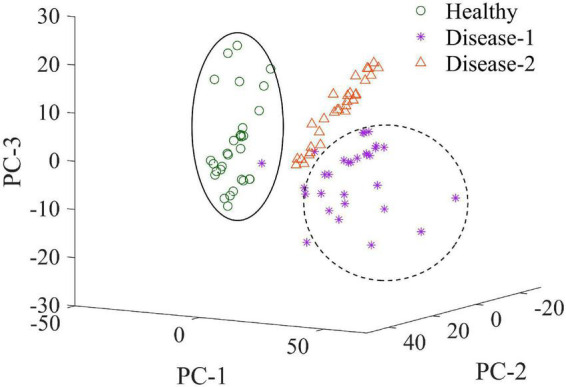
Score plots of first three PCs from PCA on spectral data of the three types of samples.

As an unsupervised learning strategy, PCA was often used to demonstrate the clustering effect based on the samples’ similarity of samples in the feature space. In the present research, PCA was performed on the raw spectra of the total sample set to visualize the distribution of healthy, disease-1, and disease-2 samples. The score scatters plot of clustering analysis were shown in Figure 8. PC1, PC2, and PC3 provided 51.74, 15.01, and 11.56% of the variations among samples, respectively. The cumulative contribution of the first three PCs achieved 78.31%. Figure 8 demonstrated that the healthy, disease-1, and disease-2 samples had obvious clustering effects. Therefore, the three types of samples had distinct spectral characteristics.

### Optimal Variables Selection

There were 1,401 variables in the SERS spectra. However, spectral data contained many non-critical variables, which might reduce the accuracy and stability of subsequent discriminant models. Therefore, selecting optimal variables was essential for better choices of discriminant models. In the present research, two strategies were used to select characteristic variables: algorithm selection (CARS combined with RFrog) and manual selection.

**FIGURE 9:**
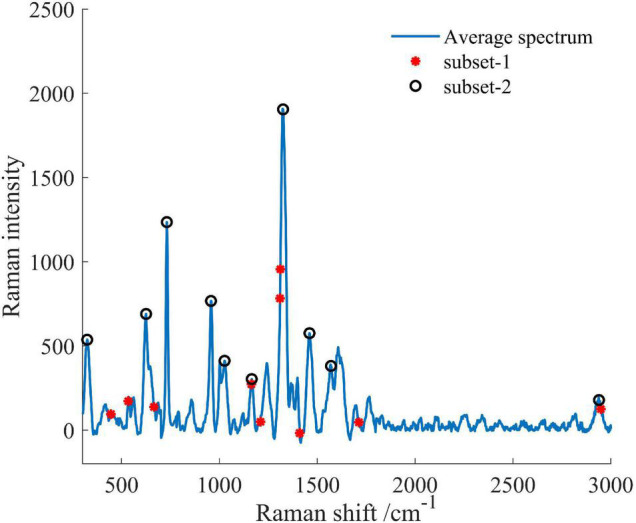
The characteristic variables for early disease detection of the AVC disease. Two strategies were used to select characteristic variables: algorithm selection (subset-1) and manual selection (subset-2).

Important variables were extracted from the total 1,401 spectral variables in the full range of 300–3,000 cm^–1^, as shown in Figure 9. The selected optimal variable subsets were set to subset-1 and subset-2, respectively. In the algorithm selection method, 10 wavenumbers at 448, 536, 667, 1,165, 1,211, 1,312, 1,314, 1,412, 1,707, and 2,951 cm^–1^ in the subset-1 were identified. In the manual selection method, 10 wavenumbers at 319, 625, 731, 957, 1,026, 1,165, 1,325, 1,460, 1,570, and 2,939 cm^–1^ in the subset-2 were identified.

### Discriminant Models Establishment

Before establishing discriminant models, SERS spectral data were divided into a calibration set and a prediction set at the ratio of 3:1. Generally, the independent variable (*x*) represented the spectral matrix of samples, and labeled grades (*y*) stood for the AVC infection severities. Therefore, the labels for healthy, disease-1, and disease-2 were 1, 2, and 3, respectively. BP-ANN, ELM, RForest, and LS-SVM models were established using four variable matrices (*x*) to classify the healthy, disease-1, and disease-2 samples. These four variable matrices (*x*) included the full SERS spectra, the subset-1, the subset-2, and the predicted fluorescence baselines.

After formula calculation and experience screening, the learning rate of the BP-ANN model was set uniformly to 0.1, and the number of neurons in the hidden layers were 10, 3, 3, and 10, respectively. The number of neurons in the hidden layer of the ELM model was determined by comparing the performances of the ELM model using different numbers of neurons from 1 to 100 with a step of 1. The ELM with 34 neurons was selected as the optimal model. The number of decision trees in the RForest model was determined by comparing the model performances using different numbers of decision trees from 1 to 500 with a step of 1. The RForest with 100 decision trees was selected as the optimal model. The LS-SVM model used RBF as the kernel function, and the optimal penalty coefficient (c) and the kernel function parameter gamma (g) were obtained by a grid search procedure. Finally, the best-c was 379, and the best-g was 45.

The discriminant accuracy of the models was presented in Table 2. There were significant differences in the classification results of the four models on the full spectra dataset. The classical BP-ANN model learned complex relationships between data, thus improving the analytical performance (such as high sensitivity and specificity) of classification. However, the BP-ANN model had the regrettable tendency to train toward a local optimal rather than a global optimal (Lussier et al., 2020). This also explained why the BP-ANN model had the lowest classification accuracy on the full spectra dataset compared to the other three models. As opposed to the BP-ANN model, the LS-SVM model was deterministic and its solution was global and unique. As a result, the classification accuracy of the LS-SVM model improved significantly compared to the BP-ANN model. In the present case of the RForest model, each tree selected features maximize the separation of the dataset into three classes. The output of each decision tree was then pooled, leading to the final optimal classification result. Therefore, the RForest model also exhibited excellent analytical performance comparable to the LS-SVM model.

**TABLE 2:** The discriminant accuracy of the models.

Models	Discriminant accuracy (%)				Running time (s)
	Full SERS spectra	Subset-1	The subset-2	Predicted fluorescence baselines	
BP-ANN	86.22	93.17	92.42	91.80	0.28
ELM	92.36	85.35	88.93	95.39	0.01
RForest	98.46	96.67	95.87	99.57	0.15
LS-SVM	98.86	94.49	95.48	98.04	0.91

Compared with the full spectra dataset, over 99% of non-critical input variables (10 vs. 1401) were removed in subset-1 and subset-2. Meanwhile, the classification accuracy of the subset models was not decreased significantly, which demonstrated the superiority of the optimal variable selection strategies. Generally, the fluorescence baselines reduced the simplicity and robustness of a calibration model built on the raw spectra. The existing studies by other scholars had removed the fluorescence baseline from the raw data. However, the classification accuracy of the models based on the fluorescence dataset was surprisingly excellent in the present research. When infesting the phloem tissue, fungus *Valsa mali* produced various chemical substances such as protocatechuic acid, isocoumarin, and phlorizin. Although these chemical substances produced fluorescence interference, the baseline reflected the chemical composition and content information. Thus, the fluorescence baseline became available as valid information. This innovative discovery will guide our subsequent research.

However, the above three methods mainly focused on feature extraction, optimal parameters, and optimal variables selection without considering the model runtime, which was also crucial for intelligent online detection, were not investigated. Furthermore, the intelligent online detection would be an important research direction in plant disease detection fields. The ELM model randomly generated the hidden node parameters and then analytically determined the output weights instead of iterative tuning (Huang et al., 2006). Thus, the ELM model runs quickly and lends itself to real application scenarios, which is very important for intelligent online detection. As seen in Table 2, the ELM model ran as fast as 0.01 s, far better than the other three methods. The LS-SVM model first used the grid search method to select the best-c and best-g, severely delaying the discriminatory efficiency and making the run time as high as 0.91 s. Therefore, the ELM algorithm can be considered as the detection model in the subsequent online detection study.

### Chemical Imaging Analysis of the Disease-Health Junction

**FIGURE 10:**
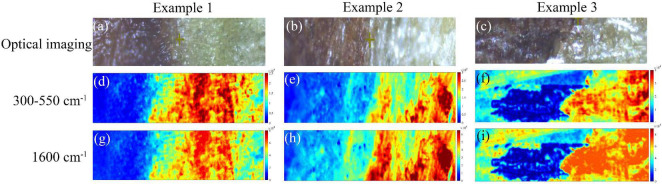
Chemical distribution images of phloem samples based on SERS spectroscopy. **(a–c)** The optical micrographs of the phloem tissues taken from the branches of three trees. **(d–f)** The chemical imaging results based on the cellulose signature peak at 300–550 cm^–1^. **(g–i)** The chemical imaging results based on the lignin signature peak at 1,600 cm^–1^.

The SERS micro-spectral image data cube of each phloem sample was processed by the air-PLS algorithm to eliminate fluorescence baseline, and the parameter values were consistent with section “Overview of Surface-Enhanced Raman Scattering Spectra.” Then the processed micro-spectral cube in a pixel-wise manner generated chemical distribution images in Figure 10. The symmetric tensile vibration at 1,600 cm^–1^ in lignin was identified as the characteristic peak of lignin components, while the bands at 300–550 cm^–1^ were contributed by cellulose. Therefore, these images were constructed based on the cellulose signature peak at 300–550 cm^–1^ and lignin signature peak at 1,600 cm^–1^.

Due to the fact that cell walls were probed in phloem tissues, the spectra collected did not contain any intracellular signals. Figures 10a–c showed the optical micrographs of the phloem tissues taken from the branches of three trees. The chemical imaging results based on the cellulose signature peak at 300–550 cm^–1^ were shown in Figures 10d–f. The redder-colored the pixels, the stronger the spectral signals of the chemical component. Meanwhile, the bluer-colored the pixels, the weaker the spectral signals. It can be noticed that the SERS signal at the healthy tissue exhibited high intensity with red, bright yellow, and green pixel colors. The diseased phloem tissue exhibited low intensity with blue and green pixel colors, and the disease-health junction exhibited green pixel colors. These differences in SERS imaging of different regions can be attributed to differences in cell wall components. The chemical imaging results based on the lignin signature peak at 1,600 cm^–1^ were shown in Figures 10g–i, showing a similar pattern as the cellulose distribution. The different regions of the phloem tissue shown a distinct distribution of cellulose and lignin, and the observations here were in good agreement with optical micrographs. The results suggested that cellulose and lignin in the cell walls of infected tissues reduced significantly. It also confirmed previous research (Ke et al., 2013) that cell wall degrading enzymes were considered to play an important role in fungal infection. Therefore, Raman microimaging was capable of detecting AVC at early infection stages. It is worth noting that Raman microimaging can visualize the intensity and distribution of components of the cell walls *in situ* through cytological observations. Meanwhile, this rapid and non-invasive chemical imaging strategy is superior to the other methods, such as the reagent staining method and transmission electron microscopy.

## Conclusion

In this study, SERS spectroscopy combined with chemometric methods was applied for early detection of the AVC disease. Firstly, three spectral preprocessing algorithms were compared, and the air-PLS algorithm was considered effective in removing the spectra fluorescence background. Thereafter, PCA provided a good clustering effect to visualize the distribution of samples in three classes. Two strategies selected optimal variables to develop machine learning models for detecting AVC disease, and these models exhibited excellent analytical performance. Meanwhile, the classification accuracy of the models based on the fluorescence dataset was surprisingly excellent, which was a great inspiration. Besides, this study proposed a new strategy for SERS chemical imaging of the diseased apple phloem tissues using a non-destructive, label-free method. This chemical imaging provided the spatiotemporal dynamic characteristics of changes in the cellulose and lignin of the phloem disease-health junction under fungus stress, which would be helpful in the early AVC detection and analysis of disease dynamics.

## Data Availability Statement

The original contributions presented in the study are included in the article/supplementary material, further inquiries can be directed to the corresponding author/s.

## Author Contributions

SF: writing – original draft and writing – review and editing. JL: investigation, resources, writing – review and editing, and revision. YW, FZ, and YZ: investigation and resources. KY: conceptualization, investigation, resources, and writing – review and editing. All authors contributed to the article and approved the submitted version.

## Conflict of Interest

The authors declare that the research was conducted in the absence of any commercial or financial relationships that could be construed as a potential conflict of interest.

## Publisher’s Note

All claims expressed in this article are solely those of the authors and do not necessarily represent those of their affiliated organizations, or those of the publisher, the editors and the reviewers. Any product that may be evaluated in this article, or claim that may be made by its manufacturer, is not guaranteed or endorsed by the publisher.
